# Advances in toxicology and medical treatment of chemical warfare nerve agents

**DOI:** 10.1186/2008-2231-20-81

**Published:** 2012-11-28

**Authors:** Mohammd Moshiri, Emadodin Darchini-Maragheh, Mahdi Balali-Mood

**Affiliations:** 1 Department of Pharmacodynamy and Toxicology, School of Pharmacy, Mashhad University of Medical Sciences, Mashhad, Iran; 2 Medical Toxicology Research Center, School of Medicine, Mashhad University of Medical Sciences, Mashhad, Iran; 3 Student Research Assembly, Mashhad University of Medical Sciences, Mashhad, Iran; 4 Department of Clinical Toxicology, Imam Reza Hospital, School of Medicine, Mashhad University of Medical Sciences, Mashhad, 91735-348, I.R., Iran

**Keywords:** Nerve agents, Chemical warfare agent, Organophosphorous compounds, Pesticides, Sodium bicarbonate, Magnesium sulfate, Iran

## Abstract

Organophosphorous (OP) Nerve agents (NAs) are known as the deadliest chemical warfare agents. They are divided into two classes of G and V agents. Most of them are liquid at room temperature. NAs chemical structures and mechanisms of actions are similar to OP pesticides, but their toxicities are higher than these compounds. The main mechanism of action is irreversible inhibition of Acetyl Choline Esterase (AChE) resulting in accumulation of toxic levels of acetylcholine (ACh) at the synaptic junctions and thus induces muscarinic and nicotinic receptors stimulation. However, other mechanisms have recently been described. Central nervous system (CNS) depression particularly on respiratory and vasomotor centers may induce respiratory failure and cardiac arrest. Intermediate syndrome after NAs exposure is less common than OP pesticides poisoning. There are four approaches to detect exposure to NAs in biological samples: (I) AChE activity measurement, (II) Determination of hydrolysis products in plasma and urine, (III) Fluoride reactivation of phosphylated binding sites and (IV) Mass spectrometric determination of cholinesterase adducts. The clinical manifestations are similar to OP pesticides poisoning, but with more severity and fatalities. The management should be started as soon as possible. The victims should immediately be removed from the field and treatment is commenced with auto-injector antidotes (atropine and oximes) such as MARK I kit. A 0.5% hypochlorite solution as well as novel products like M291 Resin kit, G117H and Phosphotriesterase isolated from soil bacterias, are now available for decontamination of NAs. Atropine and oximes are the well known antidotes that should be infused as clinically indicated. However, some new adjuvant and additional treatment such as magnesium sulfate, sodium bicarbonate, gacyclidine, benactyzine, tezampanel, hemoperfusion, antioxidants and bioscavengers have recently been used for OP NAs poisoning.

## Introduction

Chemical warfare nerve agents (NAs) are one of the important groups of organophosphorous (OP) compounds that have been used as tactical weapons and for terrorism during recent decades. OP compounds have also been used as petroleum additives and pesticides [1]. Although NAs are strongly similar in chemical structure and biological function to many OP pesticides, fatality potency of NAs is generally higher than the OP pesticides [2].

The NAs are traditionally classified into two classes of G and V agents, but also GV compounds (GV:*2-dimethylaminoethyl-(dimethylamido)-fluorophosphate*) which contained structures of both G and V agent are now exist. The G agents include Tabun (GA; *ethyl N, N-dimethylphophoramidocyanidate),* Sarin (GB; *2-fluoro-methylphophoryloxypropane*), Soman (GD; *3-fluoromethyl-phosphoryloxy-2, 2-dimethyl-butane*) and Cyclosarin (GF; *fluoro-methylphophoryloxycyclohexane*). The important warfare V agents include VE (*S-2-diethylaminoethyl O-ethylethylphophonothioate*), VM (*2-ethoxy-methylphosphoryl sulfanyl-N,N-diethylethanamine*), VG (*2 diethoxyphosphorylsulfanyl- N,N-diethylethanamine*), VR (*Russian VX; N,N-diethy-2-methyl-2-methylpropoxy phosphorylsulfanylethanamine*) and VX (*S-2 diisopropylamino O-ethylmethylphosphonothioate*) [3-5]. There are no common names for other G and V agents. VX is the main and oldest agent of V series which has been produced in large quantities [1,5,6]. Recently a new type of NAs has been claimed to develop named "Novichoks" (means “newcomer” in Russia). This has been attracting increasing attention in recent years, particularly among non-governmental organizations (NGOs). It has been claimed that the toxicity of certain “Novichok” agents may exceed that of VX. The action mechanism is also dissimilar to the other NAs and thus conventional antidotes may be ineffective. Though, to date, there is nothing in details on such chemical has ever been declared in the literature [7,8]. NAs are delivered by missiles, bombs, spray and cluster spray [9].

NAs have fatal effects in acute phase of poisoning and also have considerable long term complications due to irreversible inhibition of Acetyl Choline Esterase (AChE). They have been known as the most lethal agent among chemical warfare agents (CWA) [1,10-13].

It is important to consider the management of civilian casualties due to the possibility of NAs use in terrorist attacks. Despite early treatment and the use of urgent countermeasures (atropine and oxime) in exposure zones, it may take a long time to recover from or even alleviate the complications of NAs exposure. Thus, it was aimed to comprehensively explain clinical manifestations and recent advances in treatment of chemical warfare NAs poisoning in this review article.

## History

NAs were first synthesized in 1854 but were not used as a CWA in a large scale until eight decades later [13]. The G agents were first produced in Germany at IG Frben industries by Dr. Gerhard Schrader team in 1930s. They synthesized tabun in 1938 and then sarin. These compounds were named after him and his two co-workers. The letter G for G agents means German [4,14]. The V agents were synthesized after the World War II in the United Kingdom in 1952. The V agents were derived from the word victory; the share of allied forces from World War II [4,6]. NAs had not ever been used on the battlefield until Iran-Iraq war. During the Iran-Iraq conflict in 1983–1988, NAs were infamously used by Iraqi military against Iranian troops and even civilians. Among CWA, Sulfur Mustard and NAs (sarin and tabun, specifically) had been mostly used by Iraq in several chemical massacres [1].

Tabun was the first NAs used in the war at Majnoon Island in February 1984. Several thousands were poisoned by tabun and more than 300 victims died within 30 min. Mortality rate was much more in first years of the war because of the unavailabilities of protective equipment first-aid medications such as atropine and oximes auto-injectors [4,9,15]. Later in 1987 and 1988 another NA named sarin was used against Iranian troops and innocent people in Halabjah massacre [15,16]. It was estimated that over 100,000 individuals were poisoned by chemical attacks during the Iran-Iraq war. Meanwhile, NAs are associated with higher mortality than the other CWAs and had a drastic role in Iraqi missile attacks during the Iran-Iraq war [4].

Other tragedies of NA attacks were Sarin terrorist attacks in 1994 in Matsumoto, Japan and six months later in Tokyo Subways which poisoned 6,100 people including rescue staff with 18 mortalities. These terrorist incidences significantly raised interests in other countries and leaded to a number of symposia as the seminar on responding to the consequences of chemical and biological terrorism held at Bethesda, Maryland, in July 1995 [17]. United States have had two conflicts with Iraq during 1991 and 2003 which in both wars none of the countries used CWA. Iraq admitted possession of NAs to the USA in 1995 as well as other biologic and chemical weapons. In 1995, the USA also signed the Chemical Weapon Convention. According to this, all the nations that stored CWAs including NAs had to destroy their stockpiles by 2012 [5,18]. The Organization for Prohibition of Chemical Weapons (OPCW) is now responsible to control the CWA threat worldwide. Fortunately, in recent conflict and terrorist events, such as the 11Sept attacks in New York and Washington, Bali island of Indonesia, London and Madrid tube bombings, CWA was not used at all. Nevertheless, the use of CWA, particularly NAs is still a threat.

## Chemical structures and properties

**Figure 1:**
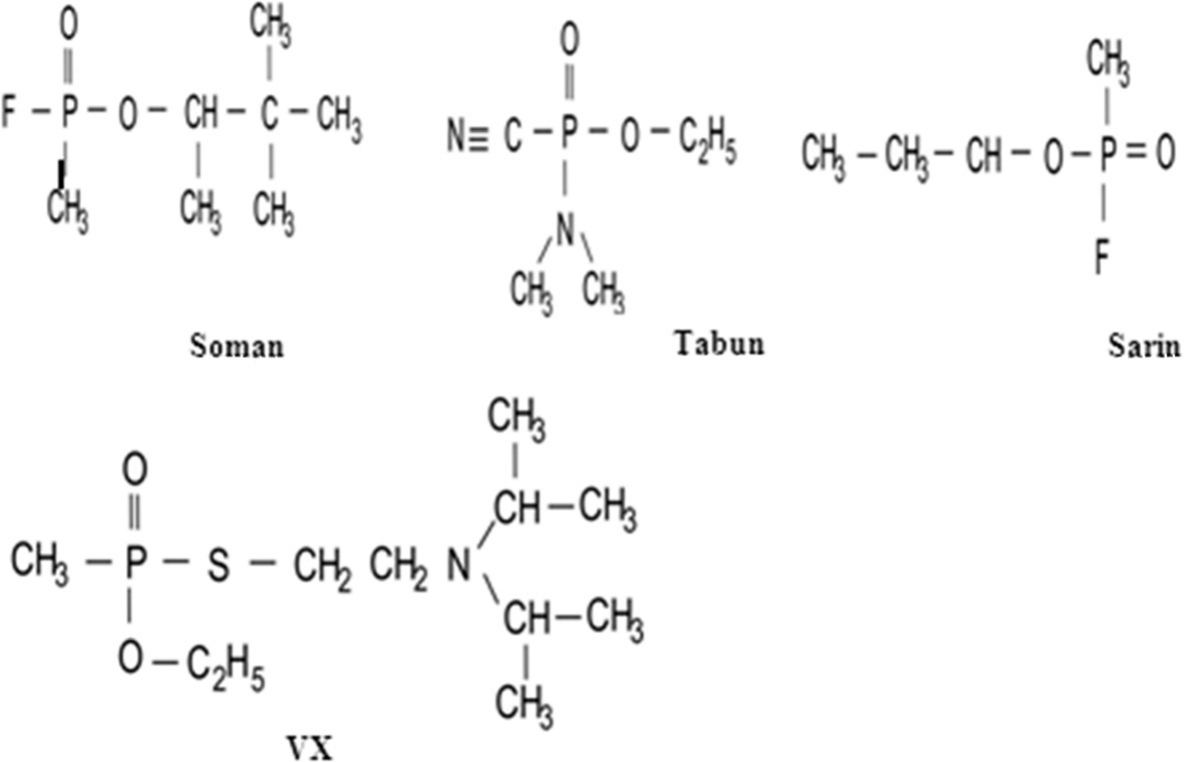
**Structural formulae of main nerve agents[**[1]**]**.

NAs are alkylphosphonic acid esters. Tabun has a cyanide group. Sarin and soman are methylphosphonofluoridate. They contain a fluorine substituent group. These NAs have a unique C–P bond that it is not found in OP pesticides and is very hydrolysis resistant. VX contains sulfur and is an alkylphosphonothiolate [19]. The toxicity of these agents are largely more influenced by the chirality around the phosphorus atom than the P(+) isomers. The structural formulae of some main NAs are shown in Figure 1 and some main properties of NAs are presented in Table 1.

**Table 1:** Physical and chemical properties of main organophosphorous nerve agents

**Properties**	**Tabun (GA)**	**Sarin (GB)**	**Soman (GD)**	**VX**
Chemical name	ethyl N, N-dimethylphophoramidocyanidate	2-fluoro-methylphophoryloxypropane	3-fluoromethyl-phosphoryloxy-2, 2-dimethyl-butane	S-2 diisopropylamino O-ethylmethylphosphonothioate
CAS No.	77-81-6	107-44-8	96-64-0	50782-69-9
Molecular weight	162.1	140.1	182.2	267.4
State	liquid	liquid	liquid	oily
Odor	Slight fruity odor	None	Slight fruity odor	None
Appearance	Clear colorless; tasteless;	Clear colorless; tasteless;	Clear colorless, ages to brown	Amber color, tasteless
Density (liquid, g/ml)	1.08	1.09	1.02	1.0083
Density (vapor, compared to air)	5.6	4.8	6.3	9.2
Volatility (mg/m3)	610	22,000	3,900	10.5
Solubility (in water, g/100g)	9.8	Miscible	2.1	Miscible<2.4 °C
Solubility (in other solvents)	Soluble in most organic solvents	Soluble in all solvents	Soluble in some solvents	Soluble in all solvents
Boiling point (°C)	220-246	158	167-200	298
Flash point (°C)	77.8	NR	121.1	158.9
Melting point (°C)	−50	−56	NR	−39 (calculated)

CAS, Chemical Abstract Service.

Unless noted, properties are determined at 25°C and pressure of 760 mmHg.

Adapted from references 6, 11, 43 and 88.

The term “nerve gas” is a historical misunderstanding, because all the classic forms of NAs are liquid at room temperature. The first chemical warfare agents (CWA) such as chlorine and phosgene are the reason as they are true gases at standard pressure and temperature [9,11]. All the NAs are liquid in room condition, tasteless and odorless and potentially volatile. However, there are some differences in chemical and density properties (Table 1). G agents’ densities are the same as water and they also evaporate at about the same rate. The freezing points are around 0°C (the same as water) and the boiling point is around 150°C. G agents rapidly spread on skin. They spread rapidly and remain in the environment for several hours and thus are known as “non-persistent agents”. G agents are released from clothing for about 30 minutes after vapor contact [12]. In contrast, V agents specially the VX are more oily, the same as motor vehicle oil and thus evaporate more slowly which is known as “persistent agents” [10-12]. Sarin is the most volatile agent with a vapor pressure of 4,000 times more than VX as of the least volatile agent [20]. Although VX has less vapor hazard than G agents (due to the least volatility), when it comes to "persistency", it can contaminate an area for longer time. Due to the oily condition, VX is the most efficiently absorbed NA through the skin [13]. Thickening agents, like acrylates, can be added to some NAs. They alter part of the physical properties of the new combined component, raising persistency of NAs in the environment [21].

Sarin is water soluble in any ratio and water hydrolyzes it to remove fluorine and produce a nontoxic product compared with the parent compound. Soman and tabun are solved easily in organic solvents, however, moderately mixed with water. VX is slightly water soluble in room temperature. Cold water and organic solvents are strong solvents for VX. G agents are quickly hydrolyzed at alkaline pH solutions. The half-life of sarin in water (pH=7.0) is 5.4 hours while it is 15 min. at a pH of 9.0. Decontaminating with alkaline solution like household bleach solutions (0.5% sodium hypochlorite) is done based on this property [22].

The vapor density of all the NAs is more than one. It means the vapor of NAs are heavier than air and they tend to stay close to the land, thus it would be a risk for people in lower areas and underground shelters [13,21].

## Mechanism of action

The main mechanism of action is irreversible inactivation of AChE at the cholinergic synapses leading to accumulation of toxic levels of acetylcholine (ACh) at the synaptic junctions. It over stimulates the cholinergic pathway and consequently desensitizes the cholinergic receptor site. ACh is a neurotransmitter which contributes to nerve conduction in central nervous system (CNS), at autonomic ganglia including parasympathetic postganglionic synapses and sympathetic preganglionic synapses. They also act at the parasympathetic nerve endings like those at neuromuscular junction of skeletal muscles and in the sweat glands [1,4,13].

There are three types of cholinesterase in human body. The main and principal form is AChE which is referred to as “true cholinesterase” and found in neurons, neuromuscular junctions and erythrocyte membrane. AChE is a serine protease that hydrolyzes the neurotransmitter ACh. It is also reported that AChE has some non-hydrolyzing functions. Park S (2004) has stated that AChE has a critical role in the development of apoptosome, a large quaternary protein structure formed in the process of apoptosis, in the body through blocking the interaction between apoptotic protease-activating factor-1(APAF1) and cytochrome C [23]. Butyrylcholinesterase (BChE) or pseudocholinesterase may have a role in cholinergic neurotransmission, and is occupied in other nervous system functions. It is also important as a biomarker of exposure to OP [24]. BChE inhibition by NAs seems to have no important physiological effect in the absence of other toxicants [25].

Serum cholinesterase (SChE) is the third form. It is a circulating plasma glycoprotein synthesized in the liver including group of enzymes present in cerebrospinal fluid, liver, glial cells and plasma. SChE does not seem to have any physiological function [4,26].

NAs play their role by binding to serine residue at the active site of AChE molecule and form a phosphate or phosphonate ester [9,11]. Thus, the resulted phosphorylated molecule is incapable of hydrolyzing ACh, and regenerate very slowly. The inhibition will be permanent until the generation of a new enzyme or a reactivator usage such as an oxime [27]. Binding reactions of NAs to esterases such as ChE, AChE, carboxylesterases (CarbE) and other proteins will also occur. Both OP pesticides and NAs lose their acyl radicals in addition to their reaction with the esterases. After binding to AChE and BChE, there is a non-enzymatic time-dependent intra molecular rearrangement which leads to loss of one alkyl group bound to the phosphorus, known as “aging reaction” (The time between NA exposure and irreversible phosphorylation). This leads to a persistant non-reactivable AChE, resistant to the both spontaneous and oxime-induced reactivation [28-30]. The half time of aging varies from a few minutes for soman, five hr. for sarin, 22 hr. for cyclosarin and more than 40 hr. for tabun and VX [9,28,31,32]. Due to reversible binding of soman and sarin to CarbE, there is a hypothesis which supposes a role for CarbE in metabolic detoxification of these agents to their non-toxic metabolites isopropyl methylphosphonic acid (IMPA) and pinacolyl methylphosphonic acid (PMPA) [31-34].

Cholinergic inhibition is not the only mechanism of action of NAs. There are some data showing other probable underlying mechanisms during NAs intoxication. Fonnum and Sterri (1981) reported that toxic effects of soman is due to only 5% of LD50 in rats, about 5μg/kg, which reacts with AChE and the rest lead to various metabolic reaction [35]. It has also been stated that NAs can inhibit enzymes outside of the cholinergic system, mainly serine esterase. It has been reported formerly that NAs alter the persistence and metabolism of some neuropeptides degraded by serine esterase, such as enkephalins, endorphins, and substance P. This may describe some atropine resistant symptoms of NAs [36]. Clement and Copeman (1984) reported longstanding analgesia in mice after exposure to sarin and soman, and nalaxone, an opiate antagonist, alleviates this phenomena. Nevertheless, no exact information is available in opioid receptors following NAs exposure [36]. Duysen and colleagues (2001) studied other probable mechanism of VX on knockout mice. They treated with 0, 50, and 100% AChE activity mice with subcutaneous VX. AChE−/− presented the same cholinergic signs of toxicity as the wild type mice, even though AChE−/− mice have not any AChE whose inhibition could lead to cholinergic signs. It was thus concluded that toxic effects in NAs exposure is due to inhibition or binding to several proteins, only one of which is AChE [37]. Other involved mechanisms included changes in other enzymes, neurotransmitter, anaphylactoid reactions, immune changes, oxidative stress etc [32,38-41]. Long lasting effects have more reasons beside ChE inhibition. It is formerly reported that NAs also act as secretagogues and can augment bronchial spasm by anaphilactoid reactions. Apart from the cholinergic crisis in NAs poisoning, secondary adverse reactions due to other underlying mechanisms may complicate NAs toxicity. Excitatory amino acids are also involved in both OP pesticides and NAs poisoning. Adenosine receptor agonists have been showed to have good protective activity on this basis [42].

## Relative toxicity

Regardless of route of entery, VX is the most potent among NAs. The stability, resistance to detoxification and environmental persistency of VX are higher than the other NAs. It is also less volatile and more impressive at skin penetration. Hence, VX is labeled as a skin penetrant and lethal contact agent rather than inhalation threat [43]. VX at 10μM largely reduced cell metabolism within two hours [44]. The G agents are toxic or even fatal by any route of exposure at sufficient concentration.

Based on animal studies, the G agents have lethal inhaled dose of about 1 mg in human. They also represent a skin contact hazard through contaminated cloths, especially when evaporation is minimized. However, the G agents absorption rate is much less rapid in percutaneous than in the inhalation form [1]. VX is easily absorbed through the skin and generally does not have a major inhalation hazard in the zone [1,43]. Overall relative lethality of NAs in animal studies is: VX>Soman>Sarin>Tabun [45].

## Metabolism

NAs’ metabolism is mostly explained under mechanism of action. The common NAs have an asymmetric center (chiral compounds), which they have two (e.g., sarin) or four enantiomers (e.g., soman) with different toxicity effects on human. Unfortunately, the more toxic enantiomers have longer half life than others in the body. Enzymatic and chemical catalysis of NAs results in the formation of inactive phosphonic acids, which are excreted via renal [46,47]. In in-vitro studies, the elimination half-life of G agents was rather shorter than V agents (less than one hour), whereas VX persists for several hours in intravenous administration and even longer in percutaneous exposure [48].

Oxidation and hydrolysis are principal metabolic reactions which occur mainly by reaction with glutathione and also may happen by glucuronidation and demethylation. Oxidation gives rise to production more or less toxic products. Tabun causes the largest number of degradation products among G agents. Detoxification of tabun takes place slowly, by the enzyme di-isopropyl-fluorophosphatase; formerly termed tabunase [49]. There are sparse toxicity data available for subset of tabun degradation products. Ethyl-dimethylaminophosphoric acid (EDMPA) is the main product of tabun dimethylamin, which is also produced by hydrolysis of tabun among other reactions. Dimethylamin cause human irritation in the respiratory tracts [50].

Isopropyl-methylphosphonic acid (IMPA) is metabolite of Sarin which subsequently hydrolyses to the high stable methylphosphonic acid (MPA) and resistance to further hydrolysis. MPA mildly irritates rabbit’s skin and human skin and eyes. It also produces low oral toxicity in mice and rats [24,51].

In rats, 10 minutes after intravenous sarin, about seventy percent of the plasma level was bound to large protein molecules similar to carboxylesterase [52]. The toxicity of sarin enhanced six to eight time when rats were pretreated with triorthocresyl phosphate (TOCP), a weak anti-ChE OP with irreversibe blocking carboxylesterase property [53].

In a study of Little *et al*. (1986) 80 μg/kg of sarin was injected intravenously to mice. Tissue distribution was recorded for 24 h. Within 1 minute sarin concentration was at the highest in the kidney, liver and plasma. Over the first minute, about half of the labeled sarin was associated with the major sarin metabolite; IMPA and the kidneys contained the highest concentration of sarin and its metabolites. Much lower concentration detected in liver after 24 hr, suggested the main role of the kidneys in detoxification of sarin [54]. In another study of Little *et al*. (1988) with the same method, hypothalamus contained concentration of both sarin and metabolites 2–5 times greater than those in other brain areas. This finding suggests that hypothalamus is important with respect to central effects of NAs [55]. Brain distribution of sarin was detected in 4 of 12 victims who died after the Matsumoto event. In patients of the Matsumoto with sarin exposure the levels of IMPA and MPA correlated with clinical manifestations [56].

Pinacolyl methylphosphonic acid (PMPA) is the predominant hydrolytic product of the soman [57].

The anticholinesterase mechanism of action of V agents is due to the "oxo" group (O) as well as presence of alkyl substituents. VX, as a V agent, is different from G agents in both pharmacodynamics and pharmacokinetics characteristics. It distributes in blood as protonated amine. Its hydrolysis is slower than G compounds and reacts more slowly with A-esterases and CarbE. Oxidation reactions at nitrogen and/or sulfur are another routes for VX metabolism beside hydrolysis [4,24]. Tsuchihashi *et al.* (1998) detected both EMPA and 2-(diisopropylamino-ethyl) methyl sulfide in VX exposed serum samples [58]. These results clarified the first documented detection of the specific VX metabolites in victims’ serum and also explained a part of metabolic pathway of VX in human body which has been later used in measuring the VX-inhibited AChE hydrolytic product EMPA [59,60].

## Detection and determination methods

Most research on diagnostic methods of NAs exposure has been directed at the most available samples of survivors such as blood (serum, plasma, whole blood, or red cells) and urine. Intact G agents are available in the organism for a few hours; therefore, blood sampling should be obtained in a few hours after OP exposure. Thus intact agents don’t seem to be a good target of retrospective detection of exposure [60-62]. There are about four approaches to detect exposure to NAs:

### AChE inhibition measurement

Although this method is the most common way to identify NAs exposure, there are some impediments in this procedure. Firstly, it does not identify the exact exposed agent and also its specification is low, because there are some other chemicals contribute to inhibition of AChE. Secondly, inhibition levels less than 20% are not detectable and it cannot be used as a retrospective measurement due to new synthesis of the enzyme. However, it is the most widely used method for evaluation of OP NAs exposure [30,60]. Wang and co-workers (2008) have assessed salivary ChE enzyme activity by using carbon nanotube-based electrochemical sensor. An electrochemical sensor based on a carbon nanotube (CNT)-modified screen-printed carbon electrode and coupled with a microflow injection system was applied for a sensitive, rapid, and simple assessment of salivary ChE enzyme activities of rat. The method provides a noninvasive biomonitoring of contact to OP NAs [61].

### Determination of hydrolysis products in plasma and urine

Rapid elimination of intact OP causes that OP-modified enzymes and metabolites are more stable in the organism. Thus, the new methods for identification and quantification of OP biomarkers modifications need to be developed [62]. Analytical methods employed are often based on gas chromatography–mass spectrometry (GC-MS), which derivatized substances before analysis, and liquid chromatography-mass spectrometry (LC-MS) which has advantage of not require derivatization.

Minami *et al.* (1997) detected sarin product MPA in Tokyo subway attack victims’ urine, using gas chromatography (GC) with flame photometric detection (GC-FPD) [47]. The GC-FPD can be useful for estimating the exposure level to sarin and is appropriate for a large number of samples.

Lately, a LC-tandem MS method has been developed for quantitative determination of IMPA in blood and urine. The main disadvantage of using hydrolysis products in NAs exposure detection is rapid elimination rate of these products (a few days) from the organism that restrict their usage in retrospective measurements [60]. John H (2010) presented matrix-assisted laser desorption/ionization time-of-flight mass spectrometry (MALDI-TOF MS) method for detecting and identifying novel adducts of human serum albumin and suggested the method as a confirmation tool for high-dose exposure to NAs [63,64] . Tabun presents a problem as its initial hydrolysis product, EDMPA and ethyl phosphorocyanidic acid, are not stable and hydrolyze further to ethyl phosphoric acid and then slowly to phosphate. Unfortunately, the general population has a high level of ethyl phosphoric acid, due to plasticizers and pesticides [65]. Several assessment methods of NAs metabolites which were mostly founded on GC-MS and LC-MS released over the past two decades. The trend is toward LC-MS nowadays and also MS-MS, which generally provides lower limits of detection than single-stage MS, and combined with a greater selectivity.

### Fluoride reactivation of phosphylated binding sites

This method is an analysis of phosphylated binding sites of BuChE in plasma or serum sample. It is based on reactivation of phosphylated enzyme with fluoride ions. The BuChE has a half-life of 5–16 days and abundant enough for biomonitoring exposure to OPs and NAs (plasma concentration, approximately 80 nMol). In this way, the extent as well as the origin of the toxicity can be determined. The other benefit of this method is ability of BuChE inhibition measurement at levels much less than those which can be measured based on decreasing AChE activity [64,66]. An analogues method was performed for the Tokyo subway sarin exposure based on isolation and trypsinization of inhibited ChE, subsequent treatment with alkaline phosphate, followed by isolation, derivatization, and GC-MS analysis of the released phosphyl moiety [67].

### Mass spectrometric determination of cholinesterase adducts

Straightforward isolation of adducted BuChE from plasma is carried out by means of affinity chromatography with a procainamide column. It is followed by pepsin digestion and LC-MS-MS analysis of a specific nonapeptide, containing the phosphonylated active site serine. This method surpasses the priors since it can also deal with aged phosphonylated BuChE [68].

A review article by Robin Black published in 2010 provides details of bio-analythycal methods for NAs detection [65].

## Clinical presentations

The complexity and persistent nature of NAs induce several organic complications among poisoned patients. Compared with other OP compounds and CWA such as sulfur mustard, NAs have relatively more acute lethal toxicity and are known as lethal agents and the deadliest CWAs [5,69]. Severity of clinical manifestations are affected by many environmental factors such as temperature, humidity, wind direction, personal protective equipment, activity level of the soldier and the time during soldier remains in the zone [69,70]. Despite primary treatment and use of urgent countermeasures (atropine and oxime), it may take long to recover or even alleviate the complications. Clinical manifestations can be divided into acute and late complications.

## Acute effects

The NAs are fatal in acute phase of heavy exposure. Thus, life threatening complications should be considered by clinicians. Depression of respiratory and vasomotor centers in the brain can induce life threatening manifestations and may lead to respiratory failure [71-73]. Hypoxia is also a life threatening effect which may lead to cerebral edema, convulsions, and histopathological brain damage [4].

ACh accumulation at the muscarinic and nicotinic receptor sites is the reason of most systemic complications. Initial symptoms and signs are mostly related to local effect rather than systemic toxicity.

### Ocular system

The most common sign in the eye is miosis which is more observed in vapor exposure. The miosis duration is varied from several days to as long as 9 weeks [74,75]. Sharp or aching ocular pain is due to ciliary spasm and can be associated with headache [76]. Impaired visual acuity, tearing and bloodshot appearance, due to subconjunctival vascular dilation, are other common features [13,73,76].

### Respiratory system

Rhinorrhea is generally considered as a local irritation effect but can also occur due to the systemic toxicity. Rhinorrhea is always heavier than those caused by hay fever or cold and the severity is dose-dependent [77]. Bronchorrhea, wheezing, bronchiolar smooth muscle constriction, and ventilator failure may be seen due to large exposure to vapor of OP NAs [73,77].

### Cardiovascular system

The expected effect is increased vagal tone which leads to bradychardia and atrioventricular block. In fact, the heart rate can actually increase. It may be due to accumulation of ACh in sympathetic ganglia and at the adrenal medulla, or because of fear and anxiety of the patient. Ventricular arrhythmias are rare [78]. Ludomirsky et al. (1982) reported Q-T prolongation in 14 and malignant tachy-arrhythmias in 6 patients out of 15 accidental OP poisoned patients [79]. Tabun, sarin and VX at 5 to 10 times LD50 caused circulatory arrest a few minutes after apnea in non-treated guinea pigs. Histopathological studies suggesting myocarditis have been reported in animal experiments, though, not conclusively proved in human studies [80].

### Nervous system

High doses of NAs can cause fatigue, muscle weakness, and even flaccid paralysis. Generalized fasciculation can continue more than other acute complications [77]. Jalali *et al.* (2011) studied patients with moderate and severe OP pesticide poisoning 10–210 days post exposure by means of electromyography (EMG) and nerve conduction velocity (NCV). On EMG, sensory-motor peripheral polyneuropathy was observed which a distal sensory deficit was predominantly. The Dysfunction of Sensory nerve (84.4%) was significantly higher than motor nerve (18.7%). Sensory nerve dysfunction in the lower extremities was more common than motor nerves, which was mainly a distal sensory deficit [81]. Seizure is also recorded as an acute effect which can be prolonged with status epilepticus. Apnea may happen abruptly and does not resolve without antidotal therapy [6]. Victims of low dose NAs exposure may experience headache, dizziness, restlessness, anxiety, mental confusion, ataxia, irritability, insomnia, bad dreams, depression, forgetfulness, impaired judgment, and lack of concentration even in absence of any physical signs [1,4,6].

### Skin and mucosal membrane

Systemic signs and symptoms can occur about two to three hours after exposure via skin. However, Skin penetrating powers of NAs are different. VX is absorbed through skin nearly eight times more rapidly than other NAs. NA skin absorption increases markedly as surrounding temperature rises from 18 to 46°C [1,82]. Generalized sweating is a common complication following prolonged dermal or inhaled exposure [77].

### Gastrointestinal system

Mobility and secretion of gastrointestinal system increase according to excess accumulation of ACh. Nausea and vomiting occur among the first signs followed by dermal exposure and can be due to nervous system complications. Diarrhea is an infrequent symptom. Among 111 patients examined after Tokyo sarin attack, 60.4% complained from nausea, 36.9% reported vomiting, and diarrhea was observed in just 5.4% of the patients [82]. Hyperglycemia may occur due to adrenal medulla stimulation that raises the blood concentrations of circulatory norepinephrine and epinephrine [6].

### Genitourinary system

Urinary system has a critical role in excretion of the NAs. During 24 hours, 76-100% of the radioactive substance presented in the urine of volunteers who received 32P-dimethoate orally [83]. Micturition can occur after large dermal contact and after the inhalation of considerable amounts of vapors [6].

### Intermediate syndrome (IMS)

IMS occurs 24–96 hours after exposure to organophosphate pesticides or theoretically after NAs exposure [84]. Recovery begins 4 to 18 days later. IMS is characterized by reversible weakness in proximal muscles, especially chest muscles, and cranial nerve palsies [85,86]. Although the etiology of IMS is not well defined, delayed AChE inhibition, down regulation or desensitization of postsynaptic ACh receptors, muscle necrosis, oxidative stress-related myopathy and failure of postsynaptic ACh release are some proposed involved mechanisms [87]. Plasma AChE of less than 200 units is a predictor and the 30 Hz repetitive nerve stimulation depreciatory response could be a useful marker for the IMS [88]. Because of potential dangers of IMS, clinicians must be aware of the syndrome and should perform neuromuscular studies and use mechanical ventilation if necessary. However, there is limited data regarding the occurrence of IMS after NAs exposure [89]. IMS has not been observed obviously after NAs intoxication in animal nor has it been noted in the handful of persons with high contact to NAs [90].

### Organophosphate-induced delay neuropathy (OPIDN)

It is defined by sensory and motor disorder of the peripheral nervous system 2–4 weeks after exposure and characterized by progressive weakness, impaired reflexes and distal paraesthesia [91]. Inhibition of an enzyme called neuropathy target enzyme (NTE) in CNS is responsible in OPIDN [92]. Degeneration of myeline and axons and inhibition of NTE is the probable etiology of OPIDN. After 1–4 weeks post exposure, approximately 30% of the patients represent cholinergic irritation (nose secretion, increased salivation, pharyngitis, and laryngitis) following by paralysis of the leg muscles which persists for 1–2 month but does not leave any changes in sensitive innervation. Then, denervation and atrophy of the leg muscles is observed [86,91,92].

## Late complications

The NAs are less likely to cause chronic diseases in comparison with other CWAs. However, NAs poisoning was reported to have association with late complications in both experimental animals and human beings. Hypoxic encephalopathy is one of the most remarkable long-term neurologic effects of NAs reported by Newmark [11]. Cardiomyopathy has been reported in soman and sarin intoxicated rats, which may be contributory cause of death, however it is not reported in human cases yet [80]. Neurological assessment of 43 Iranian veterans 22–27 years post exposure revealed fatigue, paraesthesia and headache as the most common symptoms and sensory nerve impairments as the most common observed clinical complication. The authors concluded that late neurological complications of CWAs poisoning are notable [93]. Sensory nerve dysfunction is more prevalent than motor nerves, which predominantly was a distal sensory deficit [94].

Engel *et al*. (2004) described fatigue as one of the presentations of “Gulf war syndrome” as well as depression and chronic pain [95]. Electroencephalogram (EEG) studies on sarin patients showed considerable slowing with bursts of high voltage waves at a rate of five per second epileptic type changes of EEG, 11 months after the exposure [96,97]. Asthenia, insomnia, fatigue, blurred vision, narrowing of the visual field, shoulder stiffness, slight fever, and asthopenia was associated with grades of sarin contact 1 and 3 years after Tokyo subway explosion [98]. Long-term psychological effects are also recorded. Fullerton and co-authors (1990) on a review of article mentioned temporary psychological effects such as depression, insomnia, fatigue, nervousness, irritability, and memory impairment as long-term complication of acute and chronic exposure to NAs [99]. Page (2003) on a telephone survey of 4,022 sarin exposed patients 28 years post exposure reported significantly more concentration lack and sleep disturbances in the patients in comparison with the controls [100]. Grauer *et al*. (2008) has studied late neuronal and behavioral deficit after sarin exposure to rats. The glial activation following neural damage was also established. The data showed long lasting impairment of brain function after single sarin exposure in rats that developing with time [101]. There is not acceptable evidences on carcinogenicity, mutagenocity and teratogenocity of NAs [32,41,76].

## Management of NAs poisoning

### First aid advices (hot zone)

Treatment for a severe NA exposure must be started immediately, and even seconds are important for making the difference between life and death. The first aid for victims of NAs is their immediate removal from the field or contaminated area. The rescuers should worn protective devices to prevent exposure. They must pay attention to Airway- Breathing-Circulation (ABC), and put the unconscious casualty in recovery position to prevent aspiration as the consequence of possible vomiting. When the victim is apneic and medical aid station is not near, anybody who wants to assist might consider mouth-to-mouth ventilation [102]. The rescuer should be sure about presence of vapor hazard before initiation, though, is not always possible. Only less than 10% of inspired sarin is expired [103]. It is estimated that hazard of expired breathing of casualty is minor [102].

It must be remembered that usually the casualties of NA attack are not pure chemical victims and they might simultaneously have other blunt or penetrating injuries that need evaluation and treatment. Thus they must be completely assessed.

As quickly as possible, decontamination and antidote therapy, based on severity, should be initiated [1]. Antidotes could be injected with several type of auto injectors by own victim or everybody finds him.

There are some types of auto-injectors that have different amount of antidotes, such as MARK I kit and antidote treatment nerve agent auto-injector (ATNAA) [102,104]. MARK I kit, which is the most popular one, is composed of 2 mg atropine (0.7 ml) and 600 mg 2-pyridine aldoxime methyl chloride (2-PAMCl) [105]. The ATNAA, designated by the Department of Defense of U.S., contains 2.1 mg/0.7 mL atropine and 600 mg/2 mL 2-PAM, and has ability of simultaneously injection both of them through single needle [102,106].

Every soldier carries 3 kits and one auto-injector containing 10 mg diazepam when there is a suspicious of NA attack [105].

One MARK I should be given to a casualty with only miosis and severe rhinorrhea. The second one should be added depends on the severity of respiratory distress. Applying three MARK I kits and diazepam is necessary when severe breathing difficulty or apnea, cyanosis, muscle fasciculation or twitching, seizure or loss of consciousness are present [105,107-110].

The dose of atropine of MARK I kit is between therapeutically desirable dose and safely administrable dose to a non-intoxicated person [102]. The major disadvantage of 2 mg of atropine is decreasing in sweating. The walking tolerance of 35 soldiers who were treated with 2 mg of atropine significantly decreased because of raising their body temperature resulted in limitation of sweating [111].

Absorption of antidotes when administered with autopens is more rapid than by intramuscular needle-and-syringe, because injection by autopens sprays the liquid throughout the muscle as the needle goes in, while the classical types of needle-and-syringe make a “globe” or puddle of liquid in muscle [102].

### Decontamination

NAs vapor readily absorb through inhalation and eye contact and they rapidly produce local and systemic effects. However, the absorption of the liquid types is readily through the skin, their effects might be postponed for several minutes and even up to18 hours [110]. Contaminated skin or clothing of victims can contaminate others by close contact or through off-gassing vapor [110]. Thus, decontamination should be performed as soon as possible to reduce skin absorption of NAs and prevention of the rescuers contamination, members of medical team and other patients [1,105]. This is the beat that all casualties before transport are decontaminated [110].

Medical and paramedical personnel who manage and assist to casualties either in the field, during transportation or in the hospital, must protect themselves from NAs contamination [105]. Eight staff who had taken apart in Matsumoto incident had mild symptoms of sarin poisoning [112]. Two important things for this purpose are applying personal protective equipment and decontamination of patients before entering a clinic or a ward [1]. Surgical or similar mask and gloves are not sufficient, and personnel should apply mask containing a charcoal filter, heavy rubber gloves and proper cloths. They should avoid skin contact with victims before decontamination [1,105,109,113].

Furthermore, patient handling equipment, such as gurney and back boards, should be decontaminated to prevent cross contamination. Because of easier cleaning of fiberglass back boards, it would rather to use this type of the equipment [110].

The decontamination has two important parts: physical and chemical. Early physically removal of the agent is more preferred than chemically. It means that decontamination should not be delayed because of unavailability of suitable solution and it must be started by the best means available such as water, soap plus water or other common household products to prevent NA absorption. In sarin contaminated animals which were flushed with water, dose requirement of Sarin for inducing the same morality rate as non decontaminated animals have been raised up to 10.6 times. In This method, physical removal predominates over hydrolysis [114].

Desirable secondary objective is detoxification (destruction chemically) of the NA. Chemical decontamination may be performed by methods: water/soap wash, oxidation, and acid/base hydrolysis [114]. G agents and VX have phosphorus groups that can be hydrolyzed [114-116]. Furthermore, VX contains sulfur molecules which are readily subject to oxidation reactions [114,115]. Oxidation/Hydrolysis is one of the main routes of CWAs decontamination [114].

Oxidative chlorination with "active chlorine” is the most important reactions of chemical decontamination. A 0.5% sodium or calcium hypochlorite solution (household bleach) for decontamination of skin followed by copious water rinsing is recommended. A 5% solution of the household bleach should be used for contaminated tools [1,114,117].

The hydrolysis rate of NAs increases at pH values higher than 8 and it also four times increases for every 10°C rise in temperature of water [118]. However, there are some potent detoxificant solutions, such as NaOH.They can damage the skin and tissues [114]. VX and the G agents are quite well hydrolyzed by alkaline pH, hypochlorite, as well as sulfur mustard [15,70,114,117,118].

Washing with water/soap, fresh water or sea water can also remove warfare agents through hydrolysis, slower than the other type of physical decontamination [15,18,119]. High lipid solubility of NAs significantly limits the hydrolysis rate [114]. Applying alkaline soap may increase the detoxification through rising water solubility and alkaline hydrolysis [4,114].

All contaminated clothes, shoes and jewelry of victim, should be taken off and flooded in a 5% solution of hypochlorite or put inside a plastic bag and sealed. Decontamination of intertriginous areas, axilla, groin, under the nails and hair, is also essential [1,120]. If the casualty only exposed to NA vapor, skin decontamination is not necessary, whereas his/her clothes should be all removed [105].

M291 resin kit, that contains carbonaceous adsorbent, a polystyrene polymeric, and ion exchange resins , is well suited for field use due to small and dry thus soldier are able to carry it easily [114,121,122]. It can be used on the skin, the face, and around wounds. As powder is scrubbed over the contaminated skin, its carbonaceous material rapidly adsorbs the agents and physically removes them from skin. Then the trapped agents in the interior of the resin particles will be neutralized through chemical detoxification due to the presence of basic and acidic groups in the resin. These groups destruct the agents by way of acid and base hydrolysis [114,123].

It seems that dry powders like flour, earth, and soap detergents are useful. In emergency condition, pushed flour over contaminated area, followed by wiping with wet tissue paper, has been efficient against soman, VX, and sulfur mustard [124].

The ability of mass production of G agent degrading enzyme is possible with over-producing recombinant cell line that has encoded genes of OP acid anhydrolases [125]. In addition, for detoxification of NAs, *Escherichia coli,* which OP hydrolase was expressed on surface, is immobilized by utilization of cell immobilization technology [126]. Phosphotriesterase extracted from the soil bacteria *Pseudomonas diminuta* is also applied for recognition and decontamination of insecticides and CWAs [127]. One BChE mutant G117H, was prepared through protein engineering techniques, can hydrolyze V and G agents, however, it does not react so fast [1]. There is some sponge made by a polyurethane matrix that is covalently coupled cholinesterases. They can trap and detoxificate NAs from contaminated surface [1,4,128].

As mentioned previously, NAs casualty are not pure chemical victims and they may suffer some types of other injuries and wounds which have or not bandage dressing and need to decontaminate. The toxicity of NAs could reach to wound tissues and increases injuries and toxicity. In this state, due to rapid absorption, a small drop could be lethal [128]. VX absorption is less quickly than other NAs and may persist longer in the wound [110]. All bandages should be removed and wounds be decontaminated by flushed water and remove all foreign materials from wounds. Wounds will be bandaged again only if bleeding recurs. Tourniquets and Splints are also replaced with clean ones, under physician supervision, and original sites should be decontaminated [13,114].

Cross contamination of surgeon works with contaminated wounds result by NAs on foreign bodies in the wound and by the thickened agents. It does not arise from off gassing. Thus, surgical personnel do not need to chemical-protective mask when there is not foreign body or the thickened agents. The surgeons and their assistants should wear a pair of thin, butyl rubber gloves or double latex surgical gloves and change them when they sure that the wound is free of foreign bodies or the thickened agents [110,129,130].

As Hypochlorite solution has potential for corneal injuries, it is contraindicated for the eyes [105]. If the eyes have been exposed to liquid NAs, they should be irrigated within minutes of exposure with running water or saline by leaning the head to the side, pulling eyelids apart with fingers, and pouring solution gentle [1,110]. However, flushing is not necessary for an eye exposure to NA vapor [110]. Hypochlorite solution is also contraindicated for irrigation of the abdominal cavity and not recommended for brain and spinal cord injuries [114]. Do not induce emesis in cases of NAs ingestion, administer activated charcoal without delay, if the victim is conscious and able to swallow [110].

## Treatment

### General

The patients should be evaluated completely because the NA poisoning may be complicated with multiple traumas or other CWAs. Conscious patients with full muscular power will require minimal care. Victims with possibility of liquid exposure need to be observed at least 18 hours [110].

Airway, breathing, and circulation should be evaluated. Administer antidotes without delay. Intubate the trachea in case of respiratory compromise and suck excessive bronchial secretions [110]. Severely hypoxic patients need supplemental oxygen with positive end-expiratory pressure [1,2]. It is important that improved tissue oxygenation before administration of atropine to reduce ventricular fibrillation risk [4].

### Anticholinergics and Atropine sulfate

Atropine is a parasympatholytic and competitive antagonism of ACh on muscarinic receptors [15,131]. It is an antidote for the muscarinic signs but not nicotinic and CNS symptoms of NAs poisonings [6]. Therefore, atropine has not been able to neutralize fasciculation, weakness, flaccid paralysis, or respiratory arrest which is resulted by blocking neuromuscular nicotinic receptors [4,132]. In addition, atropine does not restore blocked AChE and thus it is not curative [6,102]. But it is very effective in reversing bradycardia, drying the secretions of exocrine glands and reducing smooth muscle constriction result in decreasing bronchoconstriction and hyper motility of gastrointestinal [15,102,105]. The goal of treatment with atropine sulfate is, alleviation of bronchoconstriction and bradycardia and drying the secretions. Thus, its dosage should be titrated based on these aims and there is no clarified exact dose for atropine [1]. Balali-Mood has recommended the following protocol based on his experience on Iranian OP pesticides intoxicated patients and the Iranian soldiers who exposed to NAs,: Atropine is started at 2 mg, as available in auto-injector, and will be added based on patient response up to sings of mild to moderate atropinization (tongue dryness, reduced secretion of oropharyngeal and bronchial tree, tachycardia, and flushing) be appeared [1,4,15]. The same dose that induces initial atropinization should be constantly infused in 500 mL dextrose 5% to sustain mild atropinization and repeated based on need until the patient becomes asymptomatic. The main objective is the dryness of the mucosal membrane [1,4]. However, according to clinical experience of Balali-Mood, dose requirement of atropine for NAs is much lower than for the severe OP poisoning [1,4]. It may be due to greater fat solubility and slower metabolic rate of OP pesticides in compare with NAs [102].

Foroutan, who had treated Iranian causalities in the field and another hospital of Iran, reported another similar atropine administration protocol. He has recommended 4 mg atropine for initial dose. Then after 1 to 2 minutes, he administered another 5 mg intravenously over 5 minutes unless atropinization signs had presented. He checked pulse rate through infusion. He titrated his dose according to pulse rate and tried to set at 60–110 beat per minute in adults [102,133].

The endpoints of atropinization has been recommended by different authors, Balali-Mood [1,4], Foroutan [133], Eddleston [134], Sidell [102], are very similar:, ease of respiration, lack of bronchoconstriction, drying of respiratory secretions, and a heart rate > 80 beats per minute.

Atropine absorbs via bronchial tree, thus it could be administrated in hypotensive patients through endotracheal tube or intratracheally, and it will be shown local and systemic effect [1,4,15]. “Medical aerosolized nerve agent antidote” (MANAA) is an inhaled form of atropine which is used by United States military physicians and has been approved by FDA [102].

Atropine could not revere NAs induced miosis, except in high doses [102,105] and the pupils’ size is not a response indicator [102]. If 15 to 30 minutes after the vapor exposure has terminated, a victim has only presented miosis, atropine administration may not be indicated [102]. However, if victim, with only miosis, is visited immediately after nerve agent vapor exposure, he/she should receive one Mark I kit or ATNAA [102]. Ophthalmic application of atropine like hematropine could reduce severe eyes or head pain associated with nausea and the miosis. As these topical application are able to cause prolong blurred vision, they should not be used without appropriated reason such as severe pain.

Atropine Side effects include delirium, inhibition of sweating that induces heat related illness [105].

It is predictable that any compound that could block cholinergic might have antidotal activity [102]. More lipoid soluble anticholinergic substances could penetrate the CNS more readily than atropine and display greater antidotal activity [96,135].

Benactyzine is a lipid soluble anticholinergic drug which has been used as antidepressant [136,137]. Although, its administration for these indications is limited due to the side effects, it is shown that the CNS effects of NAs intoxication are reversed more rapidly by benactyzine than atropine [102,135]. Furthermore, benactyzine inhibits sweating or impairs accommodation much less than atropine. Therefore, it seems that it is more suitable than atropine for soldiers particularly in warm environments [102]. Benactyzine could also terminate NAs induced seizure more effectively than diazepam, in guinea pig model [138]. Some countries use “TAB” for immediately nerve agent treatment. It contains TM B-4 (an oxime), atropine, and benactyzine [102].

### Oximes

Oximes are mainly pyridinium compounds which are divided into mono and bi-s pyridium. Their general formula is R1R2C=NOH, where R1 and R2 represent any carbon group or hydrogen [1,139]. They are nucleophilic substances and reactivate the phosphonylated cholinesterase enzyme by breaking the nerve agent-enzyme bond [13,105]. Therefore, it is believed that Oximes are more physiologic antidotes than atropine for NAs poisoning [102,140].

According to mechanism of oximes action, it seems that they might completely reverse the NAs effects. However, there are five reasons that they are practically less effective than atropine as follows:

(I) It is possible that NAs act through mechanisms other than ChE inhibition [102,107,141].

(II) The oximes are unable to reverse apparent clinically muscarinic sings and they act on the nicotinic sites [4,15,105].

(III) Most of oximes are quaternary drugs with limited CNS penetration, thus they could not improve central effects of NAs intoxication [142,143]. Although a prodrug of 2-PAM, that is tertiary amine, has penetrated into the blood brain barrier, it quickly undergoes oxidation in the brain to produce an active 2-PAM and reactivate OP-inhibited AChE in the CNS. However synthesis of this prodrug is complicated now due to rapid autoxidation [144].

(IV) A meta-analysis results of six clinical trials on OP poisoning demonstrate a high relative risk for death among oxime-exposed [2,17]. It also showed the necessary to have ventilation of patients who received oxime was 1.53 more than others. And the incidence of IMS for patients who received oxime was 1.57 higher than patients treated without oxime . The authors of this paper concluded that oximes are not only effective in the management of OP poisoning, but also they can be dangerous and worsen the patient's clinical situation [145].

(V) When cholinesterase attached to NAs gets to be aged, it will become resistant to reactivation by oxime or water [107,139]. This reaction limits the efficacy of oxime in fast aging NAs as soman [105,107].

Efficacy of different types of oximes against NAs are not equal (Table 2) [4].

**Table 2:** Relative effects of oximes in organophosphrous nerve agent poisoning

**Oximes**	**Soman**	**Tabun**	**Sarin**	**Cycoserin**	**VX**
HI6	++++	+/++	+++	++/+++	+++
HLO7	+++	-	++++	++++	+++
HGG12	+++	-	NA	NA	NA
2-PM	++	-	++/+++	+/−	++/+++
TMB4	NA	++	NA	NA	NA
BI6	NA	-	++	NA	++
obidoxime	+/+++	++	++	++	+++
pyrimidoxime	++	++	++	+	++
K oximes	-	++/+	NA	NA	NA

NA=No data available - =no effective += less effective ++= mild effective.

+++= moderately effective ++++= most effective.

Boskovic had evaluated efficacy of HI-6, HGG-12, and paralidoxime (2-PAM) in conjunction with atropine and diazepam on soman and tabun intoxicated dogs. He reported that HI-6, HGG-12, and 2-PAM had showed the best protective effect in soman-poisoned dogs. However none of them had shown significant protection against tabun [146]. In another in vitro study, both H oximes (HLO-7, HI-6) and BI-6 were found to be more effective in reactivation of sarin and VX-inhibited AChE than 2-PAM and obidoxime. However, HLO-7 was less effective than HI-6. The HLO-7, HI-6 and BI-6 could not reactive tabun-inhibited AChE efficiently [147,148]. Reactivating potency of AChE inhibited by soman, sarin, cyclosarin, and VX is decreased in the order of HLO7>HI-6>obidoxime>2-PAM [1]. Therapy of intoxicated rat with GV demonstrated best antidotal effect of combination of benactyzine, atropine and HI-6 [149].

It is estimated that both newly developed K oximes (K074, K075) have higher efficacy in antidotal effect on acute tabun poisoning [150,151]. Some studies on tabun intoxicated mice and rats have been shown that K074 is more potent reverser tabun-inhibited brain AChE in rat than the other commonly used oximes [152,153]. Also K074 and K075 were effective in reversing tabun-inhibited blood AChE of rat almost as much as obidoxime [152,153]. Both of them have presented much more therapeutic efficacy in tabun intoxicated mice than obidoxime and HI-6 [150]. In rats intoxicated with tabun, reactivation of inhibited AChE in brain tissue was increased in the order HI-6 < K048 < obidoxime. This reactivation was prominent in frontal part and HI-6 was not a good reactivator against of tabun intoxication [154]. In another report, oxime effectiveness against tabun poisoning decreased in order Trimedoxime (TM B4)> 2-PAM > K127> K117 [102,155].

HI-6 is more effective in treatment cyclosarin toxicity of mice and reversing rat cyclosarin -inhibited AChE of blood and brain than other oximes such as obidoxime and K oximes [150,153]. Based on other studies, HI-6 and HLo7 have been extremely effective against cyclosarin, although obidoxime was fairly effective and the least effective agents were pyrimidoxime and PAM-2Cl [4].

In soman-intoxicated guinea pigs, HI-6 is slightly more effective than HLO-7 [156]. If efficient doses of HI-6 is administrated it can achieve efficient concentration in bran to reactivate inhibited AChE [1]. The signs of soman poisoning have positive correlation to AChE inhibition and negative correlation to the level of unbound HI-6 in the brain [157]. Also, brain uptake of HI-6 is significantly reduced by soman intoxication [157]. The K oximes (K074, K075) and obidoxime had no effect on reversing rat soman-inhibited AChE of blood and brain, however, HI-6 was very effective [153].

HLO-7 may reactivate phosphorylated muscular AChE by sarin, cyclosarin, soman, and tabun in decreasing order [158]. HLo-7 is extremely effective in tabun intoxicated guinea pigs in compare of HI-6 and pyrimidoxime [156].

Pro-2-PAM is a pro-drug dihydropyridine derivative of the 2-PAM. The pro-2-PAM has showed reactivation of sarin or VX-inhibited AChE of brain tissues and peripheral, in a dose-dependent manner, although it has been greater efficient in peripheral tissues compared to brain [159]. Pro-2-PAM has blocked sarin- or VX-induced seizures as well [159]. Though, Pro-2-PAM had no reactivation of cyclosarin-inhibited AChE in brain or muscle tissues [159]. This oxime also had no effect against cyclosarin-induced seizures [159].

The results reinforce the theory that therapeutic response of oximes depends on NA type [150]. Although, other factors such as cost and availability of the oxime and its side effects influence the selection of the oxime [1]. For example, toxicity of obidoxime (especially with high doses) is higher than 2-PAM and HI-6. However HI-6 is not as commercially available as obidoxime or 2-PAM in several countries [4]. While, the majority of our knowledge about side effects of oximes are limited to animal studies, the human experiences are limited to apply 2-PAM and obidoxime either in pesticides or war/terrorism [1]. In the United Kingdom the methanesulfonate salt of 2-PAM is the standard oxime, whereas, in other European countries TMB4 and obidoxim are used. Pralidoxime iodide is used in Japan. 2-PAM was chosen for use in the United States. HI-6 is used in Canada [102].

Administration of 2-PAM should be started at a dose of 30 mg/kg (up to 2 grams) intravenously over 30 minutes and followed by continues infusion of 8–10 mg/kg/hr (up to 650 mg/h) in dextrose 5% solution. It could be continued till the full healing or atropine is required [1,4,160]. Animal studies showed that a plasma level about 4 μg/mL could reverse sarin-induced neuromuscular block. Administration of 2-PAM with the ComboPen or MARK 1 auto-injector (600 mg) intramuscularly could produce a plasma concentration about 6.5 μg/mL in an average soldier (8.9 mg/kg in a 70-kg male) [102].The oximes should be initially administered after or at the same time of atropine [102,108,129].

In humans, 2-PAM adverse effects are minimal at therapeutic doses [102,160]. Transient dizziness, blurred vision, diplopia and elevations in diastolic blood pressure may be depended to the administration rate. Some of other reported adverse effects include: headache, drowsiness, tachycardia, increased systolic blood pressure, hyperventilation, decreased renal function, muscular weakness, nausea, vomiting and pain at the injection site [102,129,160]. Administration of 45 mg/kg 2-PAM can elevate systolic and diastolic blood pressure up to 90 mm Hg and 30 mm Hg, respectively [102]. The elevations may persist for several hours. Hypertensive effect could be minimized by giving the oxime more slowly (over 30–40 min) and reversed by phentolamine 5 mg, intravenously [102,160]. Rapid intravenous administration of 2-PAM has produced sudden cardiac and respiratory arrest due to laryngospasm and muscle rigidity [161-163]. Due to side effects administration of more than 2.5 g of oxime through 1 to 1.5 hours is forbidden [102].

More than 80% of 2-PAM is excreted unchanged through the kidneys within 3–12 hours [164,165]. The main suggested mechanism of 2-PAM kidney excretion is active tubular excretory mechanism [102,164,165]. Heat, exercise, renal failure and thiamine could decrease clearance and excretion of 2-PAM [102,164,165].

Initially and daily doses of obidoxime is not recommended more than 500 mg and 750 mg/day, respectively, due to its hepatotoxicity. During obidoxime therapy regular control of Liver function tests should be done [1]. Also liver enzymes concentrations must be observed in patients that receive doses of 1200 to 1800 mg through auto-injector contain 2-PAM. Enzymes concentrations return to normal within 2 weeks [160].

### Convulsion and Diazepam

The results of a study about efficacy of diazepam in treatment of NAs have shown that it would be an excellent adjunct therapy [1]. Convulsions should be controlled by utilizing diazepam (0.2 to 0.5 mg/kg in children and 5 to 10 mg in adults) [110,130].

It has not only symptomatic anticonvulsant effect but also has more specific effect on cholinergic and GABAergic systems [1]. In severe cases of NA exposure, convulsion (or what are described as “convulsive jerks” or “spasms”) starts within seconds after losing consciousness and collapsing the casualty. It will persist for several minutes until the victim becomes flaccid and apneic [102]. It is not reported that the convulsion has recurred after atropine and oxime therapy and ventilation support. In these cases, specific anticonvulsive therapy is not required [102].

In animal models, diazepam has been revealed to control NA–induced seizures/convulsions [138,166,167]. It also reduces brain lesion induced by NAs [138,168]. Also, food and drug administration (FDA) have approved diazepam for treatment of status epilepticus seizures via intramuscular route [102]. Thus, auto-injector of diazepam (contain 10 mg) as Convulsive Antidote Nerve Agent (CANA), is given to US military personal for immediate anticonvulsant treatment of NAs casualties in the field [102]. CANA is not considered for self-use, it rather uses in a soldier exhibits severe effects from a NA by others [102,169]. However, it is recommended to self-injection following the third Mark I or ATNAA, if soldier needs to receive all three kits [102].

Other anticonvulsant benzodiazepines e.g. lorazepam and midazolam, are effective in stopping NA–induced seizure [102,138,167,168,170]. Midazolam, however, is more potent and more rapid than diazepam in stopping NA-induced seizure [171]. It is recommended that midazolam replace diazepam as the urgent anticonvulsant treatment for NA-induced seizures. Barbiturates, phenytoin, and other anticonvulsants are not effective against NA-induced seizure [110].

Some anticholinergic drugs like atropine, benactyzine, aprophen, azaprophen, trihexyphenidyl, procyclidine, biperiden and scopolamine, had been tested for their ability to terminate soman induced seizure, in compare of diazepam, in guinea pigs. When drugs had been given 5 min after seizure onset, all these anticholinergic compounds except atropine, were able to terminate seizures at lower doses than diazepam. Seizures were rapidly terminated by procyclidine, Benactyzine, and aprophen. At 40 min after seizure onset, the most potent compound was diazepam that was followed by scopolamine, benactyzine and biperiden [138].

### Anti-NMDA and anti-glutamate drugs

Glutamate, as an excitatory amino acid plays a role in the continuance of OP-induced seizures through overactivation of the N-methyl-D-aspartate (NMDA) [172,173]. Some of them are listed in Table 3.

**Table 3:** New recommended treatments for organophosphorous nerve agents

**Category**	**Drug**	**Benefit**
*Anti-NMDA and anti-glutamate drugs*	Gacyclidine	Early administration could prevent the mortality
	Tezampanel	It reduced the length of status epilepticus induced by soman exposure. Useful in protection of neuropathy induced by soman
	Ketamine	Could stop seizure and reduced seizure-related brain damage, protection against OP nerve agent poisoning of peripheral and CNS AChE
	Huperzine A	Useful effects on seizures and status epilepticus prevention in post-exposure,
*Magnesium Sulphate:*		Administration in the first day decreases hospitalization period and improve outcomes in patients
*Antioxidants:*	Vitamin E	Therapeutic effects in OPs induced oxidative stress
*Bioscavengers:*	BChE purified from human plasma (HuBChE)	Therapeutic blood concentration of BChE can be kept for at least 4 days after a single dose administration
	Fetal bovine serum AChE (FBSAChE)	Protected against multiple LD50s of organophosphate NAs
	Fresh frozen plasma (FFP)	No significant effect

Gacyclidine is a novel anti-NMDA compound which was approved for human use in neurotraumatology [174,175]. In an animal study, soman intoxicated primates were pretreated with pyridostigmine and treated by atropine, 2-PAM, and diazepam. Another group received additional gacyclidine. Only gacyclidine was able to ensure complete recovery of NA poisoning for rapid normalization of EEG activity, clinical recovery and neuroprotection [175]. Early administration of gacyclidine added classical emergency medication could prevent the mortality [1]. In an animal study, it could prevent the neuropathology three weeks after soman exposure, however, it has not high CNS penetration [4]. Administration of gacyclidine at zero to thirty minutes after intoxication obtained optimal neuropathological protection [175].

Tezampanel, a glutamate receptor antagonist, which is specific for kainate sub-type receptors, had been useful against soman-induced seizures when administered one hr after exposure. It reduced the length of status epilepticus recorded by electroencephalographic in a 24 hours period after soman exposure. It also showed useful effects in protection of neuropathy induced by soman, as well [176].

Ketamine is a noncompetitive NMDA receptor antagonist. In one study, the effects of ketamine/atropine sulfate combinations were evaluated as delayed therapy in soman-poisoned guinea pigs. Ketamine could stop seizure effectively and highly reduced seizure-related brain damage when treated one hour post-challenge. Co-administration of ketamine and benzodiazepines increased its efficacy [177]. These effects of ketamine have been proved by another animal study on mice [178]. Ketamine plus atropine combinations have been revealed suppression of neutrophil granulocyte infiltration and partially suppression of glial activity as important neuroprotective effects. It could also reduce related pro-inflammatory proteins and mRNA excess and aroused by the soman poisoning [178].

As CNS toxic effects result from increased expiatory release of glutamate, neuroprotection can be implemented via anticholinergic effects [4]. Huperzine A is a naturally alkaloid found in the Firmoss Huperzia serrata [179,180]. It is a reversible AChE inhibitor, like donepezil, rivastigmine or galantamine [179-181] and NMDA receptor antagonist [182]. Huperzine A is able to cross the blood–brain barrier [183]. Huperzine A has revealed useful effects on seizures and status epilepticus prevention in post-exposure by blocking NMDA-induced excitation [4]. These properties make it useful in protection against OP nerve agent poisoning of peripheral and CNS AChE [184].

### Blood alkalinization by sodium bicarbonate

Effects of sodium bicarbonate in OPs poisoning were assessed in moderate to severe intoxicated patients [1,4,118]. The aim was to achieve and maintain the arterial blood pH between 7.45 and 7.55 [1,4,118]. After correction arterial acidosis with intravenous sodium bicarbonate solution, it is administered 3–5 mg/kg/24hr as continuous infusion until recovery or until atropine discontinued [1,4,118]. Dose adjustment is done based on regular arterial blood gas analysis.

Esteratic portion of OP molecules are hydrolysed in alkaline pH. Increasing one unit of pH is accompanied with 10 fold increasing in OP hydrolysis [185]. The arterial pH of higher than 7.50 makes the OP hydrolysis faster through hydrolysis acceleration [186].

Alkalinization of blood in pH more than 7.50 results in increasing urinary pH. It stimulates extraction of weak acids. The most of NAs and their metabolites are weak acids [187]. Administration of sodium bicarbonate helps to control the cardio toxicity through augmentation sodium pump channel [188]. It is also estimated that alkalinization facilitates recovery from OP poisoning thorough preventing the cardio-respiratory arrest, increasing the bio-availability of oximes, augmentation therapeutic activity of atropine and direct effect of sodium bicarbonate on neuromuscular functions [185].

### Ventilation

Ventilatory support is a main part of treatment of a casualty with severe respiratory compromise [102,105]. In animal studies, giving antidote intramuscularly at the onset of signs had been sufficient to reverse the effects of NAs, however, additional ventilation promotes the effectiveness of antidotes [102,105]. Although, some authors believe antidote therapy and intensive care management can reduce morbidity and mortality rate, the risk of respiratory failure or incapacitation do not avoid with the available antidotes (atropine, oximes) [1].

When NA vapor exposure is minimum and victim has mild to moderate dyspnea, it may be reversed by the administration of atropine [102]. Because of reversing bronchoconstriction caused by atropine, intubation of conscious patient with respiratory distress could be delayed [1]. Atropine could not reverse central respiratory arrest. If casualty suffers from respiratory distress and is elderly or has cardiac or pulmonary underling disease, he/she needs supplementary inhalation oxygen in addition to the antidotes. Patient with losing consciousness, generalized muscular twitching or convulsive jerks has apnotic or impaired respiration. Thus, they require appropriate respiratory support and should be intubated with assisted ventilation as soon as possible [1,102].

Increasing bronchial secretion is one reason of respiratory problem in NAs exposed victims. These secretions incline toward thickening, mucoid, and “ropy,” and could plug up the airways. Frequent suctioning and postural drainage of the airways can be helpful [102]. A large amount of secretions and broncho-constriction usually cause high airway resistance of 50–70 cm water [102,105]. Very high airway resistance results in causing some mechanical ventilators to malfunction [1]. After atropine administration, resistance decreases to 40 cm H_2_O or less, and the secretions reduce. Thus, ventilation is set up and respiratory support should be adjusted after starting atropine [102]. The NAs need to respiratory support much short than that applied for severe OP insecticide poisoning, because of higher fat solubility of OP pesticides that they tend to store more than NAs [15,102,168]. Unlike other CWAs such as sulfur mustard, chlorine and phosgene which induce pulmonary edema, intoxication with NAs may only require ventilator support for 20 min – 3 hours [102].

### Therapy for Cardiac Arrhythmias

NA intoxication could promote transient arrhythmias, however, it may happen after atropine administration in a normal subject [102]. High doses (5–20 LD50) of NAs ( sarin , soman, tabun and VX) intoxication in guinea pigs had caused an obvious sinus bradycardia and a consequent complete atrioventricular block within 1–2 minutes, followed by idioventricular rhythm, while, no ventricular tachyarrhythmias had been observed in these animals just before death. In this animal model, atropine and atropine plus oxime reversed right away sinus rhythm in animals which had sufficient respiration [72]. In contrast, treatment in animals without sufficient respiratory support, especially in tabun and soman (10LD50) poisoning, converted sinus rhythm to deleterious ventricular tachycardia through one minute after treatment [72]. However, this kind of arrhythmia has not been reported in humans [102]. Balali-Mood, based on his experience on Iranian soldiers intoxicated in Iran-Iraq war and patients with OP pesticides poisoning, prohibits physicians from administrating atropine to patients with tissue hypoxia e.g. cyanosis of lip and fingers. He advised to correct hypoxia, by clearing the airways and giving oxygen, before inducing atropinisation [1,4]. Valero has reported that propranolol could control cardiac tachycardia and ST depression secondary to large dose of atropine in a young woman who ingested organophosphate pesticide accidentally [189].

### Hemoperfusion

Evaluation of Effects of hemoperfusion (HP) via coated resin adsorbent synachrome E-5 in intoxicated dogs had been shown that HP in VX and sarin intoxication is only partially effective [190].

Yokoyama reported a 45 year old woman who intoxicated by sarin during Tokyo subway attack. She suffered serious NA poisoning with deep coma, pupil size less than one millimeter and respiratory problem. She had been treated with atropine, 2-PAM and respiratory support. She underwent hemofiltration and hemoperfusion because of insufficient response to treatment. Then she regained consciousness, her pupils were dilated and cholinesterase activity raised [191].

Following new achievement of intravenous lipid emulsion in treatment of intoxicated patient with lipophilic drugs [192], some authors express a hypothesis that the combination of intravenous lipid emulsions and charcoal hemoperfusion can apply to treatment severe OP poisoning [193]. However, some animal studies showed no significant effect of intravenous lipid emulsion against OP toxicity [194].

### Magnesium Sulphate

It has been reported that IV administration of magnesium sulfate (4 g) in the first day after admission would decrease hospitalization period and improve outcomes in patients with OP pesticides poisoning [195]. Magnesium sulfate reduces ACh release through blocking calcium channels [196]. It also reduces CNS overstimulation consequential from NMDA receptor activation and reversed the neuroelectrophysiological defects resulted in OP toxicity [197]. In addition, magnesium sulfate has the bronchodilating effect that is evaluated through widely trials in mild to severe asthmatic patients and it could relieve bronchoconstriction in a dose-dependent manner [198]. However, applying magnesium sulfate in NA casualties needs more research. Iranian experiences in treatment of acute OP pesticides poisoning, disclosed that alkalinization of blood with sodium bicarbonate and also administration of magnesium sulfate may be efficient in recovery of moderate to severe intoxication (Table 3) [1,118].

### Antioxidants

OP compounds generate nitric oxide and reactive oxygen radicals, decrease total antioxidant capacity, increase thiobarbituric reactive substances and lipid peroxidation in acute, subchronic or chronic exposure [4,9]. Vitamin E has shown therapeutic effects in OP induced oxidative stress in rat erythrocytes (Table 3) [4].

### Bioscavengers

There are three categories of the bioscavengers for the detoxification of OP compounds. (I) Those that stoichiometrically bind to OP compounds. Every organophosphate mole is neutralized by one mole of enzyme and both of them will be inactive. Cholinesterase, carboxylesterase, and other related enzymes are belonging to this category. (II) Some compounds that known as “pseudo catalytic” like those combining AChE and an oxime. Thus, in the presence of an oxime, the catalytic activity of OP-inhibited AChE happens fast and constantly. (III) Natural catalytic hydrolyze OP substances that make them nontoxic like paraoxonase, OP hydrolase and OP anhydrase (Table 3) [199].

Nowadays, researchers try to investigate proteins with biological scavengers’ activity on OP compounds which are acceptable to the FDA, and have ability to be stable in circulation for a long time. Through inactivating OPs before they able to inhibit AChE in CNS, could avoid the current antidotes side effects and will reduce the necessity of rapid administration of antidotes [199]. The criteria of an enzyme for applying as an effective in vivo treatment for OP toxicity include: (I) It should be able to react with all kind of OP NAs quickly, specifically, and irreversibly; (II) It should have a constant circulatory half-life (11–15 days) to be effective as a long acting scavenger; (III) The sufficient quantities of this substance should be easily available. (IV) It should not have immunogenic property [199].

Evaluation of BChE purified from human plasma (HuBChE) in animal models proposes that the therapeutic blood concentration of BChE can be kept for at least 4 days after a single dose administration. Its therapeutic index is about 30 and it is safe for human use and has not any tissue toxicity [199]. HuBChE could be stable in lyophilized form at temperatures 4°C to 25°C for 2 years. Immunological response to this enzyme had no interaction with second time pharmacokinetic profile based on animal models [199]. Fetal bovine serum AChE (FBSAChE) protected mice against multiple LD50s of OP NAs [1,4].

Fresh frozen plasma (FFP) and albumin has been recently evaluated for OP toxicity as bioscavenger. In one clinical trial on 56 OP poisoned patients, efficacy of four packs of FFP at the beginning of treatment was evaluated. The authors reported no significant differences between the two groups on the atropine and 2-PAM dosage, hospitalization length, mortality and clinical course [200]. In another study, administration of FFP, however, increased in pseudocholinesterase level, it made no favorable trends in clinical outcomes [201].

### Other new treatments

This interaction between soman and sarin plus beta-cyclodextrin, suggests that it could be a probable antidote against NAs [202,203]. The evaluation of beta-cyclodextrin ability to detoxify various NAs in vitro models, revealed its efficacy in decreasing order of cyclosarin>sarin>tabun>>VX. It could not detoxify VX. A biphasic detoxification reaction was revealed for Sarin; the primary phase, fast reduction of inhibitory potential and the second is a slower phase [204].

Cell migration resulted by cytokine therapy and stem cells engrafting into injured brain tissue of soman-intoxicated mice showed that cell differentiation into functional neurons [205]. However, this method does not ameliorate memory performance in these animals [206]. Cytokine treatment has also enhanced neuronal regeneration in the hippocampus [206].

Encapsulation of drugs or enzymes, as BChE in nanocarriers has been proposed to enhance the blood brain barrier crossing. It is thus hoped that more effective treatments will soon be available for severe neurotoxic effects of human OP pesticides and the NA poisonings [207].

Galantamine is a ChE inhibitor that acts centrally. It also is a nicotinic allosteric potentiating ligand and applied for treat Alzheimer's disease therapy. Galantamine is a safe and effective antidote against intoxication with NAs, including soman. In one study, it was compared with donepezil, rivastigmine, and (±)huperzine A, when administered 1–3 hours after soman administration to guinea pigs. Only galantamine could increase survival of the animals [208].

## Drug interactions

Medications including morphine, theophylline, aminophylline, reserpine, and phenothiazine-type tranquilizers may have interaction with OP NAs and thus should be avoided. Prescription of drug like procaine and suxamethonium (succinyl choline) that are hydrolyzed by the enzyme ChE should also be avoid [1].

## Treatment of High-risk groups

### Pregnant women and fetal toxicity

Fetal intoxication may happen because organophosphate NAs cross the placenta [4]. The sensitivity of fetus to OP compounds and atropine are higher than their mothers [4]. Clinical experience about pregnant women in Sardasht and Halabjah who exposed to sarin in the Iran-Iraq war, and pregnant women poisoned with OPs pesticides, discovered that mortality rate is higher in fetus than in the mothers [1,4]. Fetuses of survived sarin poisoned pregnant women have died within a few hours to a few days [4]. However pregnant women in the second and third trimesters of pregnancy intoxicated with commercial OP compounds have been successfully treated with atropine and 2-PAM and have delivered healthy newborns [209].

In pregnant women administration of atropine and oximes should be with caution and at lower doses. 2-PAM is a pregnancy category C and should be used as clinically necessary [160]. Obstetric consultation is necessary. Removing of dead fetus should be performed immediately after improving the mother clinical condition [4].

### Children

As casualties were seen during the Hallabjah massacre, children are more susceptible to organophosphate NAs and suffered higher mortality than adults [4]. Some reasons for this fact include: (I) children have lesser mass and more surface/volume ratio, (II) they have more immature respiratory system, (III) in young children the stratum corneum in the skin is immature that facilitates dermal absorption and (IV) their neurotransmitter systems are immature that makes children more susceptible to an epileptogenic stimulus [102,104].

The clinical manifestation of NAs in children may be quite different from adults. Miosis in OP poisonings of children is not so common as in adults, and also children may have lesser obvious convulsions/seizures [102].

Sensitivity of children to atropine and oximes is higher [104]. Atropine must be administered at least 0.05 mg/kg intramuscular or intravenously, and higher administration dose is up to 0.1 mg/kg in an obvious cholinergic crisis [102]. Atropine administration should be with monitoring of vital signs, especially the pulse rate. Atropine must be adjusted based on heart rate between 140–160 beat/min [1,104]. However, it is showed that young children generally well tolerated atropine overdose [104]. Loading dose of 2-PAM in children should be at 25 mg/kg, that is infused over 15–30 min. It may be followed by 10–20 mg/kg/hr to achieve a plasma concentration of >4 mg/L [4,132]. As half-life of 2-PAM in children is about twice of adults, in small children the initial dose might not be necessary to repeat as frequent as adult dose repetition [102].

In May 2003, the Program for Pediatric Preparedness of the National Center for Disaster Preparedness (NCDP) issued the first recommendations and treatment guidelines of pediatric disaster and terrorism awareness that is nationally accepted [210]. They recommended the Mark 1 Auto-injector kits that should be applied as first treatment of children with severe and critical NA poisoning, especially when intravenous therapy is impossible or unavailable [104,210]. When an accurate weight-based dosing of antidotes is not possible, symptomatic children less than one year old should be given atropine, while older children should be given atropine and 2-PAM from the Mark 1 kit [104].

### Elderly

Another high risk groups are the elderly. The elderly mortality and morbidity related to sarin poisoning in Halabjah and Sardasht during the Iran-Iraq war were higher than the others. In the elderly victims, complications and multiple system failure were more common than the other adults. Drug administration, such as diazepam, oxime and atropine also needs more caution [1].

## Experience in management of NA poisoning during the Iraq-Iran war

Majority of exposed people in Majnoon Island died within 30 minutes following respiratory failure, hypersecretion, convulsions, apnea and coma. Advance management of moderate to severe intoxicated patients had been done in medical centers in big cities followed applying first aid therapy and decontamination in the field hospital. Recorded clinical manifestations included hypersecretions, miosis, hypotension, diarrhea, abdominal cramps, nausea, vomiting, pulmonary edema, cyanosis, respiratory depression, muscle twitching, loss of consciousness, and convulsions. Hypotension and bradycardia were more recorded before atropine therapy while hypertension and tachycardia accompany with tongue dryness and mydriasis were more observed after atropinisation. Patients with cyanosis and extreme respiratory distress, who received high doses of atropine, had higher morbidity and mortality rate. In contrast OP pesticide poisoning, intermediate syndrome has not been reported with the NA intoxication.

There is not an exact statistical record of the NAs exposed individuals. It has been expected that in March 1984 more than 2000 patients with NAs intoxication (later on diagnosed as tabun) were managed. Mixed poisoning by tabun and sulfur mustard happened because the Iraqi army had used combination of them. Iraqi army also had used sarin accompanied with sulfur mustard in Halabjah massacre [1].

## Return to duty

There are various factors that influence deciding about returning a casualty of NAs to his/her duty. The most important criteria for person exposed in factory or laboratory is level of RBC-ChE activity. They should not return when activity of their RBC-ChE is less than 90% of their base line and are not symptoms free [13,102].

Deciding in military services is more complicated. Following considerations is important:

1) If exposure to NA is repeated, will the soldier be in higher risk due to the previous contact?

2) How much can the function of man be well?

3) What is the platoon necessary to the fighter? [102]

When measurements of blood AChE is not available, prediction about increasing danger from second NAs exposure of a soldier is difficult. However, the level of RBC-ChE activity is not a very useful criterion in field because

a) Most of the time it is not available in field.

b) A man with relatively mild effects (rhinorrhea and miosis ) may obviously have AChE inhibition.

c) The enzyme activity may be restored to near normal if soldier uses oxime (MARK I or ATNAA) and agent is susceptible to oxime [102].

## Prophylaxis

The first approach for prophylaxis against NAs, is keeping AChE intact (protection of cholinesterases) [211]. It is possible by simple chemical components like reversible inhibitors (if possible carbamates) that reversibly inhibit AChE [211]. Résistance of AChE to NAs inhibition will be higher after it inhibited by carbamates [96]. When AChE is restored spontaneously, it will act normally [96,211].

Pyridostigmine is a carbamates that binds to the AChE for a few hours [105]. Therefore, pyridostigmine is used as a “pretreatment” for NAs exposure [105]. It is administrated 30 mg every 8 hours [96,212]. It does not pass the blood–brain barrier and thus causes no CNS toxicity [1]. However, usage of higher doses present some of the same clinical manifestations of NAs, and the recommended doses caused irritating adverse effects in half of the man in a war zone [1]. The efficacy of pyridostigmine for prophylaxis in soman exposure has been approved. It could show no additional benefit in sarin or VX poisoning [1,105]. If standard therapy is not administered after the NAs exposure pretreatment will be ineffective [1]. Also, usage of carbamates should be discontinued after NAs exposure; otherwise, they will worsen, rather than protect against poisoning [1]. Physostigmine also has this ability, however, it is not the choice drug for pretreatment due to its toxicity at the amounts required [1,213].

The second approach for prophylaxis against NAs is "scavenger" effect. The scavengers are exogenous proteins (enzymes) that NAs bound to them and reduce the level NA in the organism [211,214]. However, this opinion can be considered as a "treatment in advance" [211].

Recombinant DNA-derived AChE is a bioscavenger which is potentially candidate for pre-exposure therapy for OPs toxicity [1]. FBSAChE protected mice from multiple LD50 of NAs [1,207]. HuBuChE was also useful in animal models as a prophylactic antidote against the fatal effects of NAs [207,214,215]. HuBuChE as a pretreatment has been demonstrated to enhance survival of intoxicated patients.

The third approach is applying antidotes used for NAs treatment [211]. Pretreatment with benactyzine + HI-6 was investigated in rats. It can restore soman induced circulatory and respiratory changes [1]. Due to the limitation of prophylactic effect of Pyridostigmine against most kind of NAs, Czech Army investigated pretreatment with a combination of drug (trihexyphenidyl, pyridostigmine and benactyzine,), have designated as PANPAL tablet, for soman or tabun poisoning [1,102,135,211]. Czech Armed Forces also have designed another prophylactic patch named TRANSANT that is transdermal patch containing HI-6 [216,217].

Prophylactic efficacy of Huperzine A in soman toxicity was compared with physostigmine in mice. The result showed a greater protective ratio for Huperzine A (2 times for Huperzine A and 1.5 for physostigmine) which was more long lasting (6 hour for Huperzine A and 90 min for physostigmine). The protective effect of Huperzine A had been followed a single injection, with no necessary for any post-challenge drug administration [183].

## Conclusion

NAs are deadliest CWA that need immediate intervention. Applying first aid kits like MARKI is important to reduce toxicity. However atropine and oximes are the main part of treatment. There are several adjuvant and additional therapies such as magnesium sulfate, sodium bicarbonate, gacyclidine, benactyzine, tezampanel, hemoperfusion, antioxidants and bioscavengers that have recently been used for OP NAs poisoning.

## Abbreviations

2-PAMCl: 2-pyridine aldoxime methyl chloride; AChE: Acetyl Choline Esterase; ACh: Acetylcholine; ATNAA: Antidote treatment nerve agent auto-injector; APAF1: Protease-activating factor-1; BChE: Butyrylcholinesterase; CANA: Convulsive Antidote Nerve Agent; CarbE: Carboxylesterases; ChE: Choline esterase; CNS: Central nervous system; EDMPA: Ethyl-dimethylaminophosphoric acid; FBSAChE: Fetal bovine serum Acetyl Choline Esterase; FDA: Food and drug administration; GC: Gas chromatography; GC-FPD: Gas chromatography with flame photometric detection; GC-MS: Gas chromatography–mass spectrometry; HuBChE: Butyrylcholinesterase purified from human plasma; IMS: Intermediate syndrome; IMPA: Isopropyl methylphosphonic acid; LC-MS: Liquid chromatography-mass spectrometry; MALDI-TOF MS: Matrix-assisted laser desorption/ionization time-of-flight mass spectrometry; MANAA: Medical aerosolized nerve agent antidote; MPA: Methylphosphonic acid; NA: Nerve agent; NMDA: N-methyl-D-aspartate; OP: Organophosphorous; OPCW: Organization for Prohibition of Chemical Weapons; OPIDN: Organophosphate-induced delay neuropathy; PMPA: Pinacolyl methylphosphonic acid; TOCP: Triorthocresyl phosphate.

## Competing interests

The authors declare that they have no competing interests.

## Authors' contributions

MM: Darfting the article and revision. EDM: assistance in drafting the article. MBM: Conception and design, supervising and revising the manuscript critically several times. All authors read and approved the final manuscript.
